# Multi-Cohort Federated Learning Shows Synergy in Mortality Prediction for MRI-Based and Metabolomics-Based Age Scores

**DOI:** 10.1007/s41666-025-00208-6

**Published:** 2025-07-30

**Authors:** Pedro Mateus, Swier Garst, Jing Yu, Davy Cats, Alexander G. J. Harms, Mahlet Birhanu, Marian Beekman, P. Eline Slagboom, Marcel Reinders, Jeroen van der Grond, Andre Dekker, Jacobus F. A. Jansen, Magdalena Beran, Miranda T. Schram, Pieter Jelle Visser, Justine Moonen, Mohsen Ghanbari, Gennady Roshchupkin, Dina Vojinovic, Inigo Bermejo, Hailiang Mei, Esther E. Bron

**Affiliations:** 1 Department of Radiation Oncology (Maastro), GROW School for Oncology and Reproduction, Maastricht University Medical Centre+, Maastricht, The Netherlands; 2 Section of Molecular Epidemiology, Department of Biomedical Data Sciences, Leiden University Medical Center, Leiden, The Netherlands; 3 Delft Bioinformatics Lab, Delft University of Technology, Delft, The Netherlands; 4 Biomedical Imaging Group Rotterdam, Department of Radiology & Nuclear Medicine, Erasmus MC - University Medical Center Rotterdam, Rotterdam, The Netherlands; 5 Department of Epidemiology, Erasmus MC - University Medical Center Rotterdam, Rotterdam, The Netherlands; 6 Department of Radiology and Nuclear Medicine, Maastricht University Medical Center, Maastricht, The Netherlands; 7 Mental Health & Neuroscience Research Institute, Maastricht University, Maastricht, The Netherlands; 8 Department of Electrical Engineering, Eindhoven University of Technology, Eindhoven, The Netherlands; 9 Department of Internal Medicine, School for Cardiovascular Diseases (CARIM), Maastricht University, Maastricht, The Netherlands; 10 Alzheimer Center Amsterdam, Neurology, Vrije Universiteit Amsterdam, Amsterdam UMC location VUmc, Amsterdam, The Netherlands; 11 Amsterdam Neuroscience, Neurodegeneration, Amsterdam, The Netherlands; 12 Department of Radiology, Leiden University Medical Center, Leiden, The Netherlands

**Keywords:** Federated learning, Age scores, MRI, Metabolomics, Mortality

## Abstract

While biological age scores have been shown to characterize aging by estimating chronological age based on physiological biomarkers, interactions between different age scores are largely unknown. To study this, large-scale multi-modal data are crucial. However, such data are scarce as population-based cohorts are generally restricted in sharing their data. Here, we employ federated learning to study the relationship between the two types of biological age scores: BrainAge based on brain MRI and MetaboAge based on metabolites. Using three large population-based cohorts, we trained a federated deep learning model to estimate BrainAge and compared its performance to models trained in a single cohort. The federated BrainAge model yielded significantly lower error for age prediction across the cohorts than locally trained models. Harmonizing the age interval between cohorts further improved BrainAge accuracy. Subsequently, we compared BrainAge and MetaboAge by performing association analysis and survival analysis for dementia and mortality prediction to further characterize both scores. The association analysis showed a weak association between BrainAge and MetaboAge, while the survival analysis indicated complementary predictive values for the mortality risk of the two scores. Federated learning has been shown to be a valuable technique for enabling the use of research cohorts that are restricted in data sharing. We conclude that BrainAge and MetaboAge act synergetically for the prediction of time to all-cause mortality, and both aging scores capture different aspects of the aging process.

**Supplementary Information:**

The online version contains supplementary material available at 10.1007/s41666-025-00208-6.

## Introduction

Understanding health in the context of aging is challenging, as aging encompasses various functional and structural changes in the body, including alterations in brain structure [1] and body metabolism [2]. As people age, heterogeneity among individuals increases, as some individuals may have greater health changes than what is common for their age. As a result, as aging processes start to become more prevalent, usually starting around the age of 40 [3], chronological age becomes less indicative of health. To address this issue, previous research introduced the concept of biological age, employing biomarkers based on physiological measurements [4–6]. Such biological age scores may help to understand health in the context of aging, reflecting different components of aging that can progress at a different pace between individuals, and can provide a reference for identifying pathological changes.

The biological age estimation methods consist of regression models optimized to predict chronological age from biomarkers in healthy aging individuals. These scores have been proposed based on various biomarkers using vastly different data modalities. In the field of neuroimaging, brain structure quantified with magnetic resonance imaging (MRI) was used to identify biological age predictors [7–11] (i.e., BrainAge). BrainAge has been shown to predict mortality [12], various age-related diseases—such as dementia [9], Alzheimer’s Disease (AD) and schizophrenia [11], and diabetes type 2 [13]—and non-aging-related diseases such as HIV [8]. In the field of metabolomics, several biological age scores have been proposed based on blood-based metabolomics [14–16] (i.e., MetaboAge). MetaboAge has been associated with cardiometabolic-related outcomes, such as diabetes and heart failure, as well as more general aging-related phenotypes, such as decline in instrumental activities of daily living and all-cause mortality [14]. In contrast to MetaboAge, MetaboHealth was also proposed, which predicts time to (all-cause) mortality instead of age [2]. While the predictive value of single brain-based and metabolomics-based age scores has been well studied, the relationships between these different biological age scores are largely unknown. Gaining a better understanding of this relationship may give insight on their added value and on how to optimally combine them to improve their predictive value.

For studying biological age scores, it is crucial that the models generalize effectively to unseen data from diverse sources. Therefore, large-scale data from multiple studies and institutes are required [17]. Additionally, current studies have proposed to use deep neural networks (i.e., [9]), which can capture complex patterns in high-dimensional data, and demand access to extensive datasets. The volume and diversity of data necessary for training are not usually owned by a single institution, and therefore, multi-cohort collaborations are essential. However, privacy and safety concerns make it difficult, often impossible, to centrally collect data from multiple cohorts and make it available to train these models.

In recent years, federated learning [18] has emerged as an approach to use sensitive data to train machine learning models while protecting privacy. Rather than training the model in a single institution (known as centralized learning), federated learning works by separately training at each institution’s local computing nodes and only transferring aggregate statistics, like model parameters, between institutions. A central server initiates the model parameters, aggregates the parameters sent back from each node after one or multiple epochs of local training on their local data, and then sends the aggregated parameters to each node. This routine is repeated until the model converges. As a result, it produces an optimized global model with knowledge of diverse local studies, which is trained over several distinct data collections without exchanging the data.

In this study, we apply federated learning over three population-based cohorts to study the relationship between the two types of biological age scores, BrainAge based on brain MRI [9] and MetaboAge based on metabolites [14]. We also include MetaboHealth [2] for comparation. The main contributions of our work are as follows:

1. We provide insight into the relation between the two types of biological age scores. Our results suggest the association between them is primarily driven by age, and they provide complementary information for time to all-cause mortality. However, only BrainAge is predictive of dementia.
2. We implement a federated learning infrastructure connecting three large-scale population cohorts and train a federated deep learning model for BrainAge prediction. To this end, pre-processing the imaging data in all cohorts employing the same pipeline was essential. We observe increased computational time, mainly driven by the desynchronization of the resource availability between cohorts.
3. We compare federated and locally trained deep learning models. The results emphasize the advantage of federated learning in developing models with better generalizability, benefiting cohorts without sufficient data to train a model. In addition, we show that optimizing a federated model may require further attention than a local model due to the heterogeneity between the cohorts’ data.

## Methods

### Data Preparation

#### Study Population

We included participants from three cohort studies that take part in the Netherlands Consortium of Dementia Cohorts (NCDC): the Rotterdam Study (RS) [19], the Maastricht Study (TMS) [20], and the Leiden Longevity Study (LLS) [21]. These three cohorts include imaging and blood sample data necessary for our analysis.

The Rotterdam Study is a prospective population-based study targeting causes and consequences of age-related diseases among 14,926 community-dwelling subjects aged 45 years and over [19].

The Maastricht Study is a prospective population-based study with a focus on the etiology of type 2 diabetes of 10,000 individuals. It comprises individuals aged between 40 and 75 years from the southern regions of the Netherlands [20].

**Fig. 1:**
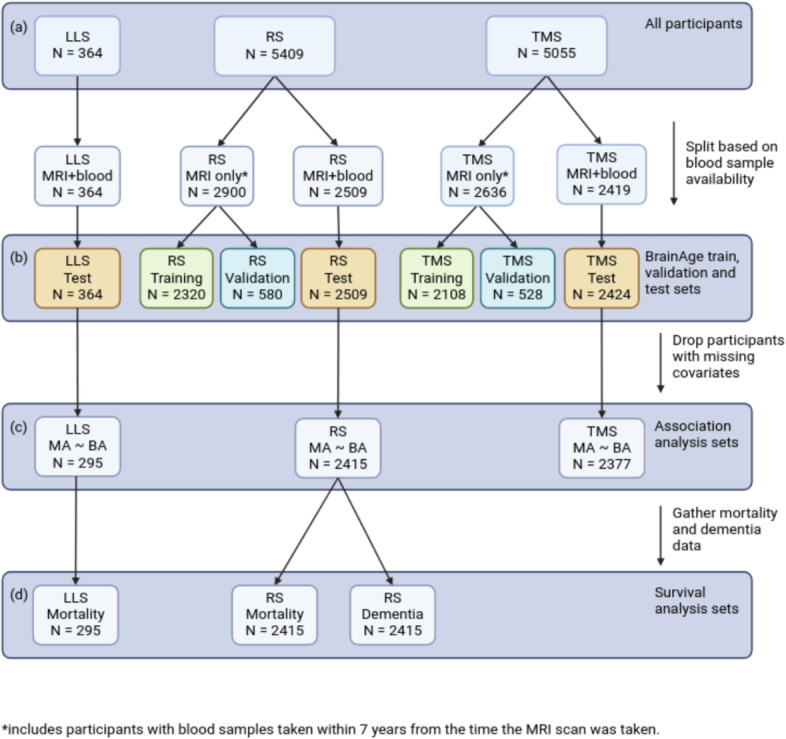
Data selection flowchart for all analyses. **a** The total number of participants included in the three cohorts: Leiden Longevity Study (LLS), Rotterdam Study (RS), and The Maastricht Study (TMS). **b** We split subsets based on the availability of blood samples for the BrainAge training. **c** The participants have both MRI scans and blood samples are used as a test set for the BrainAge model and are used for association analysis. Covariates considered are: age, sex, lag time, body mass index, diabetes mellitus, and education category. **d** We run the survival analysis on a subset of cohorts because of the availability of mortality and dementia incidence data

The Leiden Longevity study includes 421 Caucasian families, each comprising long-lived siblings, along with their offspring and the spouses of the offspring. Families meeting the criteria for inclusion had a minimum of two long-lived siblings who were alive and willing to participate. Males were considered long-lived if 89 years or older, and females 91 years or older [21].

In addition, we used data from the Alzheimer’s Disease Neuroimaging Initiative (ADNI) database (https://adni.loni.usc.edu/) in the preparation of our federated learning infrastructure (detailed in the Supplementary Material).

#### Data Selection and Splitting

The two main modalities used are T1-weighted MRI brain data [9] and metabolomic data from the Nightingale metabolomics platform measured on blood draws [14].

The data selection flowchart is shown in Fig. 1. We included participants from the studies who had at least complete data of age, sex, and brain MRI scans. For RS, 5409 participants were included from the RS-I, RS-II, and RS-III cohorts (2509 participants had both MRI scans and blood samples available, acquired within 7 years from each other). For TMS, 5055 participants were included, of whom 2419 with both MRI and blood; for LLS, 364 participants were included (all with both MRI and blood). For participants with metabolomic data, only the MRI scan taken closest to the blood sample was included for each participant, and additional covariates were selected if available: diabetes mellitus (DM), i.e., diabetes type 1 or 2 diagnosis, body mass index (BMI) and education category (EC) corresponding to their blood sampling time. Considering blood samples and MRI scans were taken at different times in RS and TMS, we used the interval years (lag time) between blood sampling time and MRI scanning time as an additional covariate.

The data was first split into two parts, based on the availability of metabolomic data. Participants with metabolomic data and absolute lag time< 7 years (between blood samples and MRI scans) were used for testing the BrainAge model, performing the correlation analysis, and the survival analysis (after excluding those with missing values in the covariates). In TMS and RS, all other participants were randomly split into training (80%) and validation (20%) sets for training the BrainAge model. LLS was used as external validation for assessing BrainAge performance on unseen cohorts. In TMS and RS, to maximize the number of training images, thereby introducing a natural type of data augmentation, we used all available longitudinal MRI scans for each participant. Table 1 shows the number of MRI scans used in the analyses.

**Table 1:** Data split in numbers of MRI scans for the three cohorts

	The Rotterdam Study (RS)$^{1}$	The Maastricht Study (TMS)	The Leiden Longevity Study (LLS)
Total number of scans	8318	5055	364
Training and validation$^{2}$	5809	2636	–
Test	2509	2419	364

$^{1}$ Includes multiple scans at different time points for some participants

$^{2}$ Includes healthy participants with MRI scans ONLY or with blood draw and MRI scan lag time > 7 years

#### Image Processing

FreeSurfer version 6.0 [22] was used to segment supratentorial gray matter (GM) based on the T1-weighted brain MRI images [23]. GM density maps were computed based on an optimized voxel-based morphometry (VBM) protocol using the FSLVBM pipeline [24, 25]. First, all GM maps were nonlinearly registered to the standard Montreal Neurological Institute GM probability template (ICBM 152 Nonlinear atlases version 2009) with a 1 $\times$ 1 $\times$ 1 mm voxel resolution. Second, a spatial modulation procedure was used to compensate for differences in absolute GM volume due to the registration. This is achieved by multiplying voxel density values by the Jacobian determinants estimated during registration. The matrix size of the modulated GM density maps was 196 $\times$ 232 $\times$ 188. As smoothing is a subgroup of possible mathematical operations which the network filters in the convolutional layer can represent, we did not apply smoothing on the VBM results. We performed a quality control based on the proportion (5%) of outlier voxels and an additional manual check to exclude the outliers. Finally, we applied cropping and padding on the images to cut proper 0 edges, and masked the images with a k-Nearest-Neighbor-classifier segmented GM mask [26]. The matrix size of the final images was 160 $\times$ 192 $\times$ 144. Image acquisition details from each cohort are described in Supplementary Material 2.1.

### Federated Learning Infrastructure

For the federated learning infrastructure, we adopted the Vantage6 Personal Health Train (PHT) framework [27]. Vantage6 is a dockerized solution for federated learning, which comes with an access control system. A Vantage6 system consists of a central PHT server node and a set of distributed PHT station nodes. Each station node is located behind the institute’s firewall, and its control connection is regulated by the central server node, which uses a private key to determine which nodes can connect. Federated learning algorithms are implemented as Docker images and authorized by each station node.

The Vantage6 framework addresses the main requirements to establish the federated network. However, additional adaptations were necessary to train the deep learning model, such as establishing a connection to local high-performance clusters and guaranteeing data interoperability between the nodes. For this, we created a technical solution by extending the Vantage6 software and harmonizing the data in each station node using a data model [28] for clinical data and the open-source Extensible Neuroimaging Archive Toolkit [29] (XNAT) for imaging data. Detailed technical descriptions can be found in Sections 1.1 and 1.3 of the Supplementary Material. Additionally, we established a governance protocol to address data security and privacy preservation, as described in Section 1.2 of the Supplementary Material.

### Deep Learning for BrainAge Prediction

We used a 3D CNN model architecture proposed by [9] to train a BrainAge model. This network takes as input the GM density maps obtained from the MRI scans and outputs a predicted age. The architecture consists of four convolutional blocks, used to extract valuable image features, followed by a fully connected layer that concatenates information on the participant’s sex. We used the mean squared error (MSE) as the loss function to train the model and optimized the model parameters based on the model with the lowest MSE on the validation set. The model’s accuracy was evaluated using the mean absolute error (MAE) on the test set. Both metrics measure the difference between model output and the participant’s chronological age. To evaluate the associated uncertainty with each model, we performed bootstrapping with resampling (1000 resamples) on the test set to calculate the 95% confidence interval. Additionally, to better estimate the model’s performance, we performed a 3-fold cross-validation.

#### Age-Bias Correction

As observed in previous studies [30], BrainAge models are prone to overestimate the age of younger participants and underestimate the age of older participants. Since this behavior can impact subsequent analysis, an age-bias correction is normally applied using a linear regression model. In our study, we calculated three age-bias correction models based on [31]’s approach, one for each training set separately (TMS and RS) and one for the federated approach. Additionally, we evaluated the generalizability of these models by assessing the performance in the cohorts’ test sets.

#### Federated Training

We trained the BrainAge model using federated averaging (FedAvg) [18]. Initially, the deep learning model weights are randomly initialized and distributed to the participating cohorts. For every cohort, the model is individually trained for several epochs on their data, starting from the shared parameters. Next, the local model is sent back to the central server. Here, the model parameters are aggregated and shared with the training cohorts. This cycle continues until reaching the convergence criteria.

#### Implementation Details

We trained the deep learning model using Tensorflow [32] (version 2.8.0) and Python (version 3.8). We did not employ data augmentation and manually tuned the hyperparameters based on the original model. Additional details are provided in Section 2.3 of the Supplementary Material. We trained the network for 20 rounds, three epochs each round, and used a batch size of eight. The model’s weights were initialized using the default Tensorflow method, the Xavier initialization [33]. The Adam algorithm was employed to train the model. Furthermore, we employed Docker [34] to containerize the scripts developed and provide the exact environment used in our experiments within a Docker image. A complete description of the libraries employed and the respective versions is provided in the public repository. Training and testing were performed at each cohort GPU cluster, specifically: an NVidia A40 GPU with 48GB and an NVidia RTX 2080 Ti GPU with 11GB for RS, a Tesla V100 with 32GB of RAM for TMS, and a TitanXp with 12GB for LLS. Finally, we followed the checklist for artificial intelligence in medical imaging (CLAIM) [35], assessment provided in the Supplementary Material, to promote the reproducibility of our work.

### MetaboAge Prediction

We applied the trained model from [14] to determine MetaboAge scores for the three cohorts. MetaboAge is a linear model based on a selection of 56 metabolites, as measured by the high-throughput proton nuclear magnetic resonance ($^1$H-NMR) metabolomics measurement platform Nightingale [36]. The original model was trained and tested on a total of 18,716 blood samples, originating from 26 Dutch biobanks with ages ranging from 18 to 85 years.

### MetaboHealth Prediction

MetaboHealth [2] is a Cox proportional hazards model trained to predict all-cause mortality. MetaboHealth uses 14 metabolites from the high-throughput proton nuclear magnetic resonance ($^1$H-NMR) metabolomics measurement platform Nightingale [36], similar to MetaboAge. These were selected using a forward-backward process that identified the metabolites with the lowest correlation with each other while being the most predictive for age at death. The original model was trained on 44.168 samples from 12 cohorts, with ages ranging from 18 to 110 years.

### The Relation Between Biological Age Scores

#### Association Analysis

We explored the association between BrainAge and the MetaboAge through linear regression. We also used MetaboHealth in the association analysis with BrainAge as comparation, since MetaboHealth is not explicitly trained on age and as such is not expected to have a high correlation with BrainAge that is driven by age. We used MetaboAge or MetaboHealth as the response variable, and BrainAge as the explanatory variable:

1 $$\begin{aligned} Y = \beta _b * BrainAge + \sum _{x_i \in X} \beta _i * x_i \end{aligned}$$

with *Y* being MetaboAge or MetaboHealth, and with *X* being the set of covariates. BainAge and MetaboAge were not corrected for age bias since we included age as a covariate in the association analysis. We first trained a model with no covariates. In addition, to correct for confounders, three sets of covariates were considered:

1. Adjustment for age: $X = \{Age\}$
2. Additional adjustment for sex, DM, and lag time: $X = \{Age, Sex, DM, Lag\ Time\}$
3. Additional adjustment for BMI and EC: $X = \{Age, Sex, DM, Lag\ Time, BMI, EC1, EC3\}$

Since age seemed to have a large effect on these models in our experiment, we also ran both 2 and 3 without age, finally resulting in 6 sets of covariates in total. All variables were normalized when training the models.

The linear regression was also performed using the federated approach. Each round *t*, the PHT server sends out global beta values ($\beta _g = \{\beta _b, \beta _1,..,\beta _i\}$) to all cohorts. These then create a local update of the beta values ($\beta _l$) using one iteration of gradient descent:

2 $$\begin{aligned} \beta _l^{t} = \beta _g^{t} - \eta * \Delta L \end{aligned}$$

with *L* being the mean squared error loss function. $\eta$ was set at 0.1 for all models. Then, each cohort sends back their own $\beta _l$ to the PHT server, which creates a new value for $\beta _g$ using a weighted average:

3 $$\begin{aligned} \beta _g^{t+1} = \frac{1}{\sum _{j=0}^J n^j} \sum _{j = 0}^J n^j * \beta ^t_{l,j} \end{aligned}$$

with $\beta ^{t}_{l,j}$ being the local beta values coming from cohort *j* at round *t*. This iterative process continued until the MAE did not change anymore. We repeated this process 10 times and chose the model with the lowest MAE. The beta values of the linear regression model were used for measuring the association of the involved covariates.

In addition, we compared the federated model to the closed-form solution using the meta-analytical framework HASE [37].

#### Survival Analysis

To assess the complementary value of biological age scores in estimating the vulnerability of individuals, we performed survival analyses using Cox proportional hazards models based on BrainAge and MetaboAge. We used the difference between participants’ age score and their chronological age, BrainAge Gap (BAG: BrainAge - Age), and MetaboAge Gap (MAG: MetaboAge - Age) as input to the survival analyses:

4 $$\begin{aligned} \lambda (t|MAG, BAG, X) = \lambda _0(t) * exp(\beta _1*MAG + \beta _2*BAG + \sum _{x_i \in X} \beta _{x_i}x_i) \end{aligned}$$

with *X* being the set of covariates adjusted for. Two sets of covariates were used: (1) age only ($X = \{Age\}$) and (2) the full set of covariates used in the association analysis ($X = \{Age, Sex, DM, Lag\ Time, BMI, EC_1, EC_3\}$). BainAge and MetaboAge were not corrected for age bias since we included age as a covariate in the survival analysis.

We compared the survival curves on the 1$^{st}$ and 3$^{rd}$ quartiles of BAG and MAG. Intuitively, individuals at this first cutoff point have a lower than average age gap, indicating that they have aged less than expected. Conversely, at the second cutoff, the age score is relatively higher than average, indicating accelerated aging. Combining the two scores, this creates four cutoff points:

- BAG 1$^{st}$ quartile, MAG 1$^{st}$ quartile: decelerated aging according to both BrainAge and MetaboAge
- BAG 1$^{st}$ quartile, MAG 3*rd* quartile: decelerated aging according to BrainAge, accelerated aging according to MetaboAge
- BAG 3$^{rd}$ quartile, MAG 1$^{st}$ quartile: accelerated aging according to BrainAge, decelerated aging according to MetaboAge
- BAG 3$^{rd}$ quartile, MAG 3$^{rd}$ quartile: accelerated aging according to both BrainAge and MetaboAge

Separate survival curves were created by applying the trained model from (4). We further examined using MetaboHealth instead of MAG, and the separate and pairwise effects of BAG, MAG, and MetaboHealth on the risks of dementia and mortality, adjusting for the full set of covariates.

**Table 2:** Population characteristics summary of 3 cohorts included in the study

	The Rotterdam Study (RS)	The Maastricht Study (TMS)	The Leiden Longevity Study (LLS)
participants with MRI scans ONLY$^{1}$	2900	2636	0
female, n (%)	1583 (54.6)	1331 (50.5)	–
age at MRI, mean (sd)	67.0 (10.5)	59.6 (9.0)	–
age at MRI, range	45–100	40–75	–
participants with MRI scans AND blood samples	2509	2419	364
female, n (%)	1433 (57.1)	1187 (49.0)	190 (52.2)
age at MRI, mean (sd)	67.5 (9.5)	60.3 (8.4)	65.5 (6.6)
age at MRI, range	46–96	40–75	45–84
participants with complete covariates eligible for association analysis	2415	2377	295
lag time (years) between blood sample and MRI scan, mean (sd)	$-$1.39 (3.22)	2.2 (1.3)	0
BMI, mean (sd)	27.3 (3.8)	26.6 (4.2)	25.3 (3.3)
educational level low/medium/high, %	46.3/29.7/24.0	30.4/28.9/40.7	55.2/8.6/36.2
diagnosis of diabetes, n (%)	221 (8.8)	538 (22.2)	21 (5.8)
diagnosis of dementia$^{2}$, n (%)	154 (6.4)	–	3 (1.0)
follow-up years of dementia, mean (sd)	6.9 (2.9)	–	13.0 (2.5)
mortality, n (%)	662 (27.4)	–	48 (16.3)
follow-up years of mortality, mean (sd)	10.0 (2.9)	–	13.0 (2.5)

$^{1}$ Additionally includes participants with blood sampling and MRI scanning lag time > 7 years, and excludes scans with dementia diagnose or stroke

$^{2}$ 8 missing values for dementia in RS and 1 missing value in LLS

The survival analyses were run locally in RS with mortality and dementia as outcomes, and in LLS on mortality only, as this cohort had only a few dementia cases at the latest follow-up ($N=3$); TMS had no long-term mortality or dementia data available.

## Results

### Population Characteristics

The subject characteristics are shown in Table 2. No demographic bias was found between participants with MRI scans only and participants with both MRI scans and blood samples available within each cohort. However, there are some biases between the cohorts. TMS has younger participants, while the RS has elder participants and a larger proportion of females. In addition, TMS has a larger proportion of diabetes cases than the other two cohorts. Such a cohort-level difference is quite common in a multi-center study, and we expect our federated learning based analysis is able to mitigate this bias.

**Table 3:** MAE [95% confidence interval] of BrainAge models trained locally (at TMS and RS separately) and in a federated way (using both TMS and RS)

		Local models		Federated model
		TMS	RS	TMS & RS
TMS	Training	3.10 [2.99, 3.23]	–	4.75 [4.61, 4.90]
TMS	Validation	4.59 [4.30, 4.87]	–	5.56 [5.23, 5.89]
TMS	Testing	4.72 [4.59, 4.87]	6.45 [6.22, 6.71]	5.59 [5.44, 5.76]
TMS	Testing $^{*}$	4.67 [4.58, 4.73]	6.55 [6.39, 6.71]	4.97 [4.84, 5.12]
RS	Training	–	2.48 [2.42, 2.54]	4.34 [4.25, 4.43]
RS	Validation	–	2.50 [2.37, 2.62]	4.87 [4.66, 5.06]
RS	Testing	7.29 [7.11, 7.44]	4.21 [4.09, 4.34]	4.36 [4.21, 4.48]
RS	Testing $^{*}$	7.00 [6.22, 7.62]	5.10 [4.95, 5.25]	4.87 [4.74, 5.01]
LLS	Testing	5.63 [5.24, 6.05]	5.66 [5.26, 6.08]	4.60 [4.25, 4.95]
LLS	Testing $^{*}$	5.82 [5.10, 6.25]	5.89 [5.51, 6.30]	4.21 [3.85, 4.60]

Models were trained and tested in The Maastricht Study (TMS) and the Rotterdam Study (RS), using the Leiden Longevity Study (LLS) as the external test cohort

$^{*}$ Testing results of the model trained with a sub-selection of the training participants with age between 53 and 75 years

### BrainAge and MetaboAge Models

#### BrainAge Model

Using the federated learning infrastructure, we ran the training and testing of our federated BrainAge model at the three cohort locations. We used TMS and RS as training cohorts, with LLS held out as an external testing cohort. As shown in Table 3, the federated BrainAge model demonstrated its capability to predict the chronological age across different cohorts with mean absolute errors (MAE) of 5.59 years in the TMS test set, 4.36 years in the RS test set, and 4.60 years in the external test set (LLS). To better interpret such federated BrainAge model performance, we also ran local models (trained at TMS or RS separately). Our federated model showed a better performance than local models that were tested on data from a different cohort. To highlight, the local model trained on RS data (training MAE = 2.48, test MAE = 4.21) achieved lower performance on TMS (test MAE = 6.45) and LLS (test MAE = 5.66) than the federated model. Similarly, the local model trained on TMS data (training MAE = 3.10, test MAE = 4.72) achieved lower performance on RS (test MAE= 7.29) and LLS (test MAE = 5.63) than the federated model, suggesting the local models were unable to maintain performance when tested with data from a different cohort. Furthermore, the performances for the federated model in each cohort and subset were more similar than those of the local models, indicating less overfitting. These observations are supported by the results obtained with the 3-fold cross-validation approach (Supplementary Table 4), indicating similar MAE estimates for the three models.

We observed that the federated BrainAge model yielded a higher MAE for the TMS cohort than for the other two cohorts. We suspected that this higher MAE was due to the TMS cohort being relatively younger than the overall training set (mean age TMS: 59.6 years; mean age RS: 67.0 years). To further validate this, we trained the federated model and two local models with a sub-selection of the participants in the training set with the same age range between 53 and 75 years in both RS and TMS (70% of the training set, mean age of 61.8 years). With this new training set with similar age ranges, we observed that the model converged to a solution with smaller MAE differences in all three cohorts (TMS test MAE of 4.97 vs 4.87 in RS and 4.21 in LLS).

**Fig. 2:**
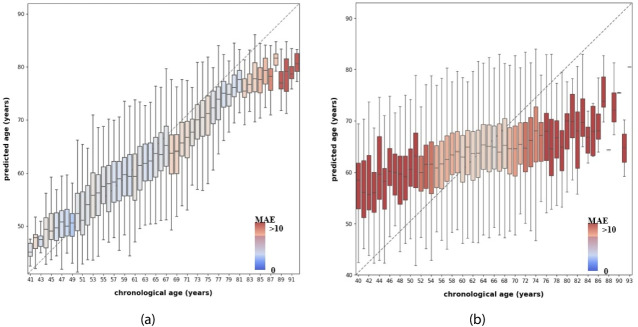
Chronological age vs. predicted age for **a** Federated BrainAge and **b** MetaboAge for the complete test set. The color indicates the predicted Mean Absolute Error (MAE) of participants at a certain chronological age, with color red indicating an MAE $>10$

Additionally, we observed a tendency of the model to overestimate the age of younger subjects and underestimate the age of older subjects, as shown in Fig. 2a. Although this tendency was apparent in all cohorts, differences existed in the age interval where it occurred between the cohorts. We therefore applied an age-bias correction to the BrainAge model (see methods Sect. 2.3.1 for details). Supplementary Table 5 shows that bias correction on the federated model resulted in considerable improvements for the RS (MAE before correction: 4.36, MAE after correction: 3.33) and LLS (MAE before correction: 4.60, MAE after correction 3.62) but little for the TMS (MAE before correction: 5.59, MAE after correction: 5.51). Moreover, evaluating the bias correction with data from a single cohort, with either the TMS or the RS training set, displayed considerable improvements in the corresponding cohort (see Supplementary figure 2, first two rows) but did not benefit external cohorts.

We further compared the computation time, which was 64% higher on average for federated training of the BrainAge model than that of a central training (Table 4). We also optimized the federated training hyperparameters, as detailed in the Supplementary Table 2. Optimized model hyperparameters were an initial learning rate of $1 \times 10^{-3}$, a learning rate decay of $1 \times 10^{-2}$, and a dropout rate in the last layer of $5 \times 10^{-1}$. In the federated architecture, selecting the model with the lowest MAE from each cohort showed optimal convergence. Performance was most similar between cohorts when models were aggregated between cohorts with equal importance weighting.

**Table 4:** Computation time (range) taken to train the BrainAge model centrally and in a federated way (same computational resources in both approaches)

Trained	Data	Computation Time (hours)	Number of Epochs	Time / Epoch (min)
Centrally	TMS	13.70 (12.93, 14.53)	100	8.22
Centrally	RS	16.83 (16.35, 17.23)	100	10.10
Federated	TMS & RS	15.22 (13.23, 16.56)	60	15.22

The results presented comprise the time from the 3-fold cross-validation training

#### MetaboAge Model

The MetaboAge models resulted in prediction performances of MAE = 7.9 for the TMS test set, MAE = 7.4 for the RS test set, and MAE = 6.4 for LLS. Overall prediction results per chronological age can be seen in Fig. 2b.

These results show a larger age bias than those for BrainAge. They suffer more from regression to the mean and have a wider range of predictions for each age bracket.

### Relation Between the Biological Age Scores

#### Association Analysis

The association analysis results between BrainAge and MetaboAge, and BrainAge and MetaboHealth are shown in Table 5. A more detailed table including p-values and standard errors can be found in Supplementary Table 7. For MetaboAge, the model with BrainAge as the only predictor (M1) resulted in a small but significant association (beta = 0.16, SE = 0.014, P = $4.3 * 10^{-32}$) (Table 5 (a)). Then, adding age as a covariate (M2) showed a strong effect on the observed relationship between BrainAge and MetaboAge (beta = $-$0.08, SE = 0.022, P = $6 * 10^{-5}$), indicating that the information both scores provide is chronological age. This effect of age on the relation between the two age scores was consistent when including sex, DM, and lag time (M3-M4), as well as when further adding BMI and EC (M5-M6). Covariates other than age did not show a strong influence on the relation between BrainAge and MetaboAge. For MetaboHealth, the association between BrainAge and MetaboHealth without covariates was similar to that of MetaboAge (Table 5 (b)). However, this correlation persisted after adjusting for age, suggesting that BrainAge and MetaboHealth share common information beyond chronological age.

**Table 5:** Beta values for various levels of covariates for estimating MetaboAge (a) and MetaboHealth (b)

(a)									
Model ID	BrainAge	Age	Sex	DM$^1$	Lag Time	BMI$^2$	EC1$^3$	EC3$^3$	Error (MAE)
M1	0.16$^*$ $^*$	–	–	–	–	–	–	–	0.77
M2	–0.08$^*$	0.32$^*$ $^*$	–	–	–	–	–	–	0.75
M3	0.25$^*$ $^*$	–	–0.17$^*$ $^*$	0.16$^*$	0.08$^*$	–	–	–	0.73
M4	–0.01$^=$	0.39$^*$ $^*$	–0.14$^*$ $^*$	0.19$^*$	0.14$^*$ $^*$	–	–	–	0.74
M5	0.22$^*$ $^*$	–	–0.27$^*$ $^*$	0.08$^*$	0.01$^=$	–0.03$^*$	0.16$^*$	0.11$^*$	0.76
M6	–0.06$^*$	0.38$^*$ $^*$	–0.23$^*$ $^*$	0.08$^*$	0.04$^*$	-0.03$^*$	0.07 $^*$	0.11$^*$	0.74
(b)									
M1	0.13$^*$ $^*$	–	–	–	–	–	–	–	0.76
M2	0.11$^*$	0.03$^=$	–	–	–	–	–	–	0.76
M3	0.13$^*$ $^*$	–	–0.04$^*$	0.70$^*$ $^*$	0.02$^=$	–	–	–	0.76
M4	0.10$^*$	0.02$^=$	-0.04$^*$	0.71$^*$ $^*$	0.04$^*$	–	–	–	0.75
M5	0.10$^*$ $^*$	–	–0.01$^=$	0.68$^*$ $^*$	0.00$^=$	0.15$^*$ $^*$	-0.07$^*$	–0.26$^*$ $^*$	0.75
M6	0.09$^*$	0.06$^*$	0.06$^*$	0.66$^*$ $^*$	0.03$^*$	0.10$^*$ $^*$	-0.10$^*$	–0.27$^*$ $^*$	0.74

$^{1}$ DM = Diabetes Mellitus, i.e., diabetes (type 1 or 2) diagnosis

$^{2}$ BMI = Body Mass Index

$^{3}$ EC1-3 = Education Category, mapped to low/medium/high based on years of education. One-hot encoded relative to the medium level

$$^{*}$$ $$P \le 0.05$$

$$^{**}$$ $$P \le 5*10^{-10}$$

$$^{=}$$ $$P > 0.05$$

#### Survival Analysis

To investigate whether BrainAge and MetaboAge have complementary information about an individual’s health, we performed a survival analysis assessing their predictive performance for the time to mortality and dementia prediction.

**Fig. 3:**
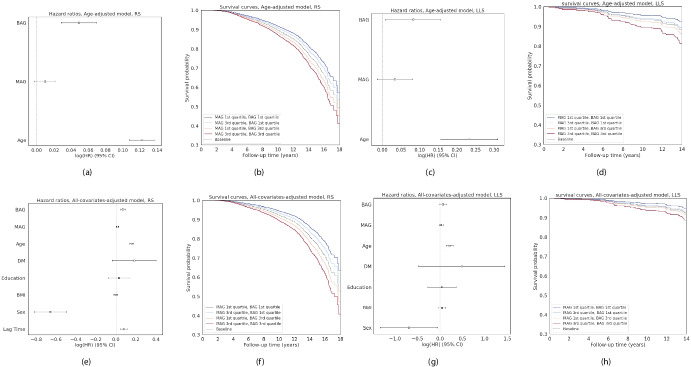
Survival analysis for mortality prediction in the Rotterdam Study (RS) and the Leiden Longevity Study (LLS) using Cox Proportional Hazard models. **a**–**d** The results of the age-adjusted models (**a** and **b** in RS, **c** and **d** in LLS), taking only BrainAge Gap (BAG), MetaboAge Gap (MAG), and age into account. **e**–**h** The results for all covariate-adjusted models (**e** and **f** in RS, **g** and **h** in LLS), additionally adjusting for diabetes mellitus, education category, body mass index, sex, and lag time. Note that the lag time in LLS is 0 for all participants

Figure 3 shows the survival curves for four groups of individuals that are either young-appearing or old-appearing as quantified by the biological age scores. Group are formed with different combinations of BrainAge and Metaboage scores, having either a relatively low or high BrainAge Gap (BAG: BrainAge-Age) and either a relatively low or high MetaboAge Gap (MAG: MetaboAge-Age). We inspected the survival of different combinations of these scores, in which a clear separation can be observed. Young-appearing individuals on both biomarkers (MAG 1st quartile; BAG 1st quartile) showed the highest survival rate, while old-appearing individuals (MAG 3rd quartile, BAG 3rd quartile) showed the lowest survival rate. Individuals scoring differently on both markers had an intermediate survival rate. As suggested by the hazard ratios of BAG and MAG, this effect was more pronounced in RS than in LLS, as both BAG and MAG have more significant effects on the survival probability in RS. For dementia prediction (Supplementary Figure 4), we found that only BAG was significantly associated with the time to dementia diagnosis, while MAG did not differentiate participants with dementia, independent of covariates. Using MetaboHealth instead of MAG in the survival analyses (Supplementary Figure 5) resulted in similar observations, though with a larger hazard ratio for MetaboHealth, likely due to the fact that MetaboHealth was trained to predict mortality.

Supplementary Table 9 and 10 show the separate and pairwise effects of BAG, MAG, and MetaboHealth on the risks of dementia and mortality, adjusting for the full set of covariates. The combined analysis, including both MAG/MetaboHealth and BAG, showed minimal changes in their coefficients and significance compared to the separate models. This pattern was consistent across both dementia and mortality outcomes, suggesting that BAG and MAG contribute independent information to the prediction of these risks. MetaboHealth, similar to MetaboAge, was not associated with dementia risk. However, it showed a much stronger association with mortality, even after adjusting for BAG. When included together in the same model, MetaboHealth attenuated the significance of MAG, indicating potential overlap in the biological information they capture regarding mortality risk.

## Discussion and Conclusion

This study demonstrated a federated BrainAge model on three large-scale population-based cohorts that outperformed local models trained on only one cohort. Our federated analysis results additionally suggest that BrainAge and MetaboAge carry non-overlapping information with regard to time to all-cause mortality.

Regarding MetaboAge results, the test performance of MetaboAge prediction was comparable to the original study with a median error of 7.3 [14]. Regarding BrainAge results, the performance of the federated BrainAge model was similar to other deep learning and 3D CNN-based models reported in the literature (MAE between 4–5 years) [38, 39]. These suggest the validity of our models. The federated model yielded significantly lower error (MAE) for age prediction based on Brain MRI across cohorts than the locally trained models, showing that the federated model has better generalizability to external data. Such generalizability is a major concern for data methods in current medical practice [40]. Although data privacy rules prevented comparing a centralized model (trained on pooled data) with the federated model, both performed similarly when trained on a publicly available dataset (Section 2.2 of the Supplementary Material). Federated learning can enable cohorts with insufficient data to train an accurate model, leveraging other cohorts’ datasets to still get accurate model predictions. However, this is only possible if federated models generalize well to unseen cohorts.

Age differences between cohorts had an impact on the results. We observed that the BrainAge model performance showed smaller MAE differences between cohorts when restricting participant selection to an equal age interval on all cohorts. Besides, we observed that both BrainAge and MetaboAge tend to overpredict the age of younger subjects and underpredict the age of older subjects, which is a known problem for biological age scores [41] based on chronological age. Part of the performance gap between cohorts can be attributed to this effect. We showed that a bias correction can help to decrease this tendency for our federated BrainAge model; however, its effectiveness varies, especially for external test cohorts. Additional methods focused on both sample-level and age-level bias may improve the correction effectiveness and the reliability of BrainAge [42]. As for explainability, Wang et al. [9] analyzed the model’s attention maps, which highlighted the importance of the amygdala and hippocampus regions in predicting brain age, especially with increasing chronological age.

Regarding the federated association analysis, we found a low association between BrainAge and MetaboAge, which was drastically reduced after adjusting for age, indicating that the main association between the two biological age scores is their common correlation with age. It is known that different aspects of aging, such as cardiovascular and cognitive decline, can occur at different paces between individuals [3]. We therefore hypothesize that the underlying reason for the low association between BrainAge and MetaboAge is that they characterize different aging aspects, while being less informative for the aspect for which the other score is most informative. The low association between BrainAge and MetaboAge suggests complementarity of both scores, which is supported by the survival analyses. In contrast, the association between MetaboHealth and BrainAge did remain when adjusted for age. This could be related to earlier observations that MetaboHealth is (like BrainAge, and unlike MetaboAge) associated with cognitive decline [43].

Aside from age, the other covariates for which we corrected had only a minor impact on the association between BrainAge and MetaboAge. These tested covariates included education level, which has a known connection with lifestyle [44, 45]. Due to low coverage, we did not adjust for other lifestyle factors such as physical activity or medication use, which could direct future research upon improving the data collection. Future study of those additional lifestyle factors will help us to better understand the observed relation between BrainAge and MetaboAge.

The survival analysis showed that individuals who scored high (indicating accelerated aging) on both BrainAge and MetaboAge had a lower survival rate than those that scored low on one or both of the scores. This suggests that BrainAge and MetaboAge have complementary information about an individual’s mortality. While only BrainAge was predictive for dementia, which also indicates the differing roles of the two age scores and the crucial role of brain-specific aging processes in the individuals’ onset of dementia.

In the survival analysis results there are specific differences between cohorts that could be explained by study design differences between RS and LLS. Mortality was lower, and BrainAge was less informative in LLS compared to RS. While LLS had inclusion criteria favoring healthy and long-living individuals, RS aims to include a general population and is less selective. We therefore hypothesize that LLS included participants with a relatively low BrainAge.

One of the limitations of our analysis was the relatively limited amount of events in our survival analysis. Survival analysis on dementia was only possible in RS due to the lack of dementia cases in LLS. The amount of cases for mortality was 27.4% and 16.3% in RS and LLS, respectively. Performing a survival analysis with more cases could strengthen our results. Another limitation is the diversity of the populations. Although RS and TMS are population-based studies, they mostly include participants from Western European descent, limiting the applicability of our findings to other populations. Finally, due to the inability to share data, our federated BrainAge model could not be compared to a centralized model trained on the same collection of data.

Federated learning and federated analysis enable the use of data for which collaboration was not possible before, thereby increasing the pool of data for research. However, using a federated infrastructure for real-life data also comes with several challenges. First, harmonized data pre-processing across cohorts is essential [46, 47]. We took account of this by harmonizing all data and reprocessing all imaging data with the same image analysis pipeline. Second, the distributed processing environment may provide challenges both for the model optimization itself as well as for the optimization time needed. Regarding optimization, data heterogeneity between cohorts can lead to either overfitting in a single cohort or fluctuation in convergence between different cohorts when training the BrainAge model in a federated setting. By altering parameters such as lowering the local amount of epochs and increasing the dropout rate, we were able to decrease overfitting on the largest cohort (see supplementary results 2.3 for details). Furthermore, we experienced that the time needed for model optimization was relatively high, as the compute resource availability was not synchronized between cohorts. Finally, Vantage6 was unable to interact directly with the cohort’s high-performance compute platform required to train the BrainAge model. Therefore, we created a technical solution by extending the station node Docker image to allow establishing a connection to local HPC platforms.

For the choice of federated learning platform, we considered two aspects. First, regarding suitability for deployment, Vantage6 provides a user authentication system and a whitelisting system for algorithms that allow secure implementation. Second, regarding the convenience of algorithm development, Vantage6 runs all algorithms in Docker containers, providing flexibility on what algorithms to use, not limited to federated learning, but also allowing, for example, federated association analysis. Nevertheless, as federated learning platforms are still emerging and under development, the choice for the most optimal platform might change over time.

In summary, this study used federated learning to train and validate a BrainAge model across three cohorts and federated analysis to perform association and survival analysis of BrainAge and MetaboAge/MetaboHealth. Our results highlight that federated learning is a promising technique for cases in which data sharing is not possible. In addition, we conclude that BrainAge and MetaboAge act synergetically for the prediction of time to all-cause mortality. We consider combining biological age scores based on different data modalities an interesting future research direction, as a combined age score will provide more complete information for understanding health and may have a higher predictive value for identifying pathological changes in individuals.

## Supplementary information

Please see the supplementary files.

## Supplementary Information

Below is the link to the electronic supplementary material.

Supplementary file 1 (pdf 3683 KB)

Supplementary file 2 (xlsx 14 KB)

## Data Availability

The data from the Leiden Longevity Study, The Maastricht Study, and Rotterdam Study are accessible for researchers upon request. Please find the required forms at: https://leidenlangleven.nl/data-access/, https://www.demaastrichtstudie.nl/research/data-guidelines. Requests for Rotterdam Study data can be directed to data manager Frank J.A. van Rooij (f.vanrooij@erasmusmc.nl). Code used to run the experiments and produce the results can be found at: https://github.com/NCDC-usecase1.

## Code Availability

Code used to run the experiments and produce the results can be found at: https://github.com/NCDC-usecase1
