# Supplementary material for: Multi-Cohort Federated Learning Shows Synergy in Mortality Prediction for MRI-Based and Metabolomics-Based Age Scores
Source: J Healthc Inform Res. 2025 Jul 30;9(4):606–28. doi: 10.1007/s41666-025-00208-6 (PMC12602841; doi:10.1007/s41666-025-00208-6)
Supplement: Supplementary file 1 — (pdf 3683 KB) [file 41666_2025_208_MOESM1_ESM.pdf]

Supplementary Material

1 Federated Learning Infrastructure

We implemented the federated learning infrastructure based on the Vantage6 Personal Health Train (PHT) framework. This framework provides a system to manage the connection between the different centers, access control, and tools to run the algorithms and retrieve the results. Generally, federated training starts when a researcher sends a request to the PHT server. In each communication round, the PHT server sends a command to the participating station nodes with information on which Docker image to run. The stations execute this specific Docker image and return the results to the central server. This server then saves these results in a database, from which the researcher can pull with their script.

1.1 Job submission to local High Performance Cluster (HPC)

Commonly, in a Vantage6 PHT system, all local jobs are executed at the station node which is often provisioned as a single virtual machine. However, this is insufficient for running the deep learning algorithms we need for BrainAge prediction with thousands of images. Therefore, we have created a technical solution by extending the station node Docker image (https://github.com/MaastrichtU-CDS/federated-brain-age/tree/master/v6_wrapper) to be able to connect to each institute's local High Performance Cluster (HPC) facility such as a SLURM cluster (at LLS and RS) or an on-premises Kubernetes managed cluster (Data Science Research Infrastructure in TMS). When a task is submitted, instead of specifying the algorithm image, a placeholder Docker image name is specified with the actual algorithm image being specified in the task's inputs. In the station node configuration this placeholder is mapped to a locally available Docker image (wrapper image). This wrapper image will be executed by the station node to redirect the task to the local HPC, download and verify the federated learning algorithm image. A local file transfer command, such as *scp*, is used inside the wrapper image to transfer the input data, task inputs and token to the cluster's file system. A job to run the algorithm image will be submitted to the cluster scheduler and this job's status will be monitored though *ssh*. Once the job has finished, the output file produced by the algorithm image will be retrieved and written to the output file as expected by Vantage6.

1.2 Security and privacy

Data security and privacy preservation is a core requirement in our analysis as defined in the project agreement so that no sensitive data (e.g., brain MRI images) will be transferred outside of a cohort intentionally or unintentionally. We have established a governance protocol to address this. As illustrated in Figure 1, first our project developers implement and jointly verify the Vantage6 PHT algorithm Docker image. Second, local institutional developers will support the cohort owners to examine these algorithm Docker images (e.g., which datasets are accessed and analyzed and which aggregated results are transferred to the server node). Third, the cohort owners decide whether to whitelist an algorithm image using its specific SHA256 digest to run on their

local node (configured through the allowed_images field in the node configuration). Any unauthorized changes in the PHT algorithm image will result in a different SHA256 digest and cause this algorithm to be denied at a PHT station. Therefore, from a cohort owner perspective, only verified and certified Vantage6 algorithms can access their local data to ensure the privacy. All data communication between PHT server and PHT stations are encrypted using RSA to further guarantee the data security.

1.3 Data management

Although Vantage6 addresses a wide range of requirements for a federated system, it does not provide an out-of-the-box solution to guarantee data interoperability between the station nodes. To address this, we harmonized the clinical data in each station node using a data model as described in [1]. Each station includes a local PostgreSQL database connected to the PHT node that guarantees structural and semantic data compatibility. Additionally, we employed this database to store the deep learning models and performance metrics. Regarding the imaging data, we homogenized the storage systems across cohorts by using the open source Extensible Neuroimaging Archive Toolkit (XNAT [2] to store the MRI scans. If not available, a central XNAT server was available for use. By storing the MRI scans in equal storage systems, the same data structure was enforced across the cohorts. After this harmonization step, we transferred the necessary imaging data to the GPU cluster for training the deep learning models, avoiding a high throughput of read and write operations.

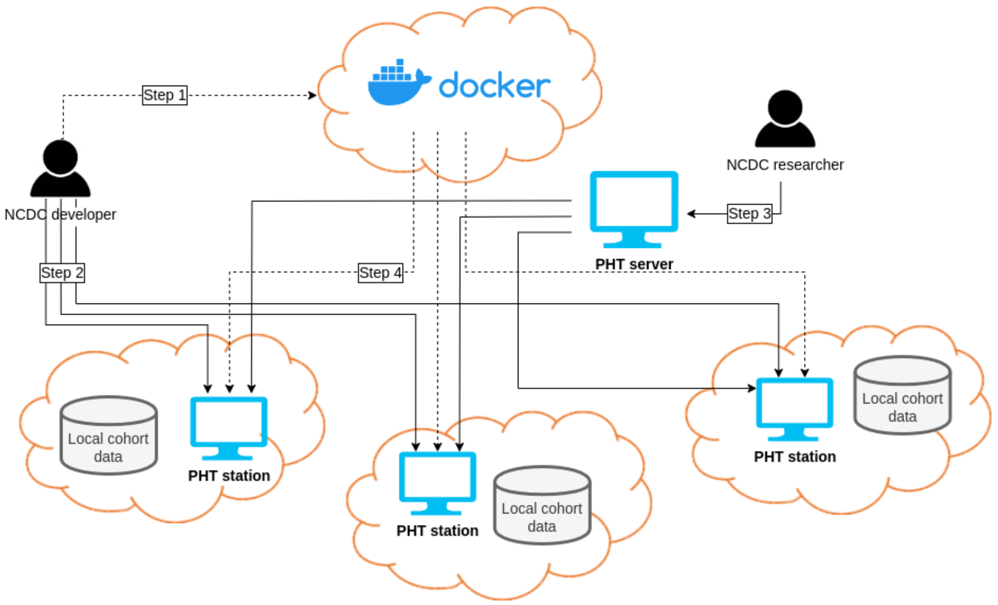

Fig. 1 NCDC PHT governance protocol to ensure security and data privacy requirements. Step 1: NCDC developers implement and verify the PHT algorithms (in Docker image). SHA256 digests of Docker images are generated. Step 2: NCDC developers support cohort owners to examine these algorithms (e.g., which datasets are retrieved and transferred), then together decide which algorithms are allowed in their local PHT station (using allowed_images tag). Step 3: NCDC researcher performs data analysis via a PHT server. Step 4: Only authorized PHT algorithms are executed at each cohort's local PHT station.

2 BrainAge

2.1 Image acquisition

Imaging data from the RS were obtained on a 1.5 T GE Signa Excite MRI scanner using an 8-channel head coil. The MRI protocol included a T1-weighted 3-dimensional (3D) Fast RF Spoiled Gradient Recalled Acquisition in Steady State with an inversion recovery pre-pulse (FASTSPGR-IR) sequence (TR = 13.8 ms, TE = 2.8 ms, TI = 400 ms, FOV = 25 × 25 cm2, matrix = 416 × 256 (interpolated to 512 × 512 resulting in voxel sizes of 0.49 × 0.49 mm2) [3].

In TMS, the data were acquired on a 3T clinical magnetic resonance scanner (MAGNETOM Prismafit, Siemens Healthineers GmbH) using a head/neck coil with 64 elements for parallel imaging. The MRI protocol included a 3D T1-weighted magnetization prepared rapid acquisition gradient echo (MPRAGE) sequence (repetition time/inversion time/echo time (TR/TI/TE) 2300/900/2.98 ms, 176 slices, 256 × 240 matrix size, 1.0 mm cubic reconstructed voxel size) [4].

Imaging in the LLS was performed on a Philips 3 Tesla Achieva MRI scanner using a standard 16-channel whole-head coil for radiofrequency transmission and reception (Philips Medical Systems, Best, The Netherlands). For each subject, a 3D T1-weighted

anatomical scan was acquired with the following scan parameters: TR = 9.7 s; TE = 4.6 ms; flip angle = 8°; voxel size 0.88 x 0.88 x 1.40 mm.

2.2 Federated V.S. centralized training

To compare the performance of the BrainAge model between a federated and a centralized setting, we conducted an assessment with publicly available data. For this purpose, we simulated three cohorts and optimized the BrainAge model hyperparameters for the federated approach. Data used in the preparation of this supplement were obtained from the Alzheimer's Disease Neuroimaging Initiative (ADNI) database (adni.loni.usc.edu)[1]. The ADNI was launched in 2003 as a public-private partnership, led by Principal Investigator Michael W. Weiner, MD. The primary goal of ADNI has been to test whether serial magnetic resonance imaging (MRI), positron emission tomography (PET), other biological markers, and clinical and neuropsychological assessment can be combined to measure the progression of mild cognitive impairment (MCI) and early Alzheimer's disease (AD).

We randomly divided the data from healthy individuals into three groups (N=221, 219, 217) and placed it in each cohort's station, simulating a federated infrastructure with three separate institutes. MRI data was pre-processed following the pipeline described in the Methods section. To evaluate the model's performance, we applied a leave-one-out cross-validation strategy by using one cohort exclusively for testing in each round. Additionally, we trained the model centrally with the complete dataset to assess the baseline performance.

The results show that the federated model converged to a solution without requiring different hyperparameters from the central model (Table 1). The BrainAge estimation from the federated learning model (average testing set MAE of 4.13) reaches similar performance as centralized learning (testing set MAE of 3.94).

In conclusion, this experiment showed that the federated model performed similarly to the model trained centrally and did not benefit from changing the hyperparameters.

Table 1 Comparative performance (MAE (range)) of the BrainAge model trained with ADNI data centrally or with the federated architecture. The results presented comprise the average MAE and range (values in brackets) from the 3-fold cross-validation.

	Training	Validation	Testing
Central model	2.22 (2.00, 2.42)	3.91 (3.55, 4.29)	3.94 (3.54, 4.34)
Federated model	2.35 (1.66, 3.67)	4.39 (4.05, 4.73)	4.13 (4.02, 4.35)

[1]The investigators within the ADNI contributed to the design and implementation of ADNI and/or provided data but did not participate in analysis or writing of this report. A complete listing of ADNI investigators can be found at: http://adni.loni.usc.edu/wp-content/uploads/how_to_apply/ADNI_Acknowledgement_List.pdf

Table 2 Performance evaluation (MAE (range)) for the federated training optimization (test set).

Weighted averaging [1]	Model selection [1]	Number of epochs [1]	TMS	RS	LLS
✓	-	3	6.71 (6.13, 7.17)	4.18 (4.04, 4.39)	5.62 (4.22, 6.85)
-	-	3	7.04 (6.30, 7.73)	4.24 (4.03, 4.44)	4.67 (4.02, 5.21)
✓	✓	3	7.53 (6.94, 8.41)	4.67 (4.02, 5.77)	4.54 (4.44, 4.67)
-	✓	3	6.34 (5.29, 7.00)	4.68 (4.53, 4.95)	4.43 (4.00, 5.16)
-	✓	6	7.92 (5.56, 9.73)	4.22 (4.06, 4.32)	4.47 (4.19, 4.70)

Table 3 Hyperparameters used to train the BrainAge models locally and using the federated approach.

	Locally	Federated
Learning rate	1×10^{-2}	1×10^{-3}
Dropout rate	0.25	0.50
Beta 1	0.90	0.90
Beta 2	0.999	0.999
Epsilon	1×10^{-8}	1×10^{-8}
Learning rate decay	1×10^{-4}	1×10^{-2}
Rounds	-	20
Epochs	100	60 (3 epochs by round)

2.3 Model optimization

The application of the federated BrainAge model to the NCDC cohorts revealed the need to further improve the hyperparameters and aggregation methods compared to the initial experiment with ADNI data. The heterogeneity of the dataset affected the model's convergence and exacerbated the problem of overfitting one of the cohorts.

To assess the impact of the model training options (number of epochs by round, model selection, and weighted averaging based on sample size), we repeated a 3-fold cross-validation for each. The results in Table 2 suggest that a smaller number of epochs, no weighted averaging, and selecting the local model with higher MAE improves the performance. Regarding the hyperparameters, we observed that a higher learning rate decay (1×10^{-2} vs 1×10^{-4}) and dropout rate (0.5 vs 0.25) benefited convergence. Table 3 provides a complete overview of the hyperparameters used.

2.4 Population differences

The performance differences observed in the BrainAge models trained locally or in a federated collaboration hinted at the possible impact of the population differences. As shown in Table 4, locally trained models display a higher MAE range in unseen data compared to the federated approach. Moreover, Figure 2 highlights this challenge by indicating different age intervals per cohort where the model underestimates and overestimates the chronological age. Consequently, applying a linear correction to the BrainAge predictions appears to be a cohort-specific solution. In addition, we

compared the model's performance between subgroups discretized by sex and diabetes diagnosis (Table 6). We did not observe substantial discrepancies between male and female participants or between participants with and without diabetes. Slight MAE differences between subgroups, as observed for participants with diabetes in TMS, can be attributed to shifts in age distribution and the reduced effect of age-bias.

When applying a bias correction to the federated BrainAge model (Table 5), it resulted in notable improvements for the RS and LLS (MAE of 3.33 and 3.62 vs 4.36 and 4.60) but little for the TMS (MAE of 5.51 vs 5.59). Moreover, evaluating the bias correction with data from a single cohort, with either the TMS or the RS training set, displayed considerable improvements in the corresponding cohort (MAE of 3.66 for TMS and 3.00 for RS) but did not benefit external cohorts (MAE of 6.31 for TMS and 7.41 for RS).

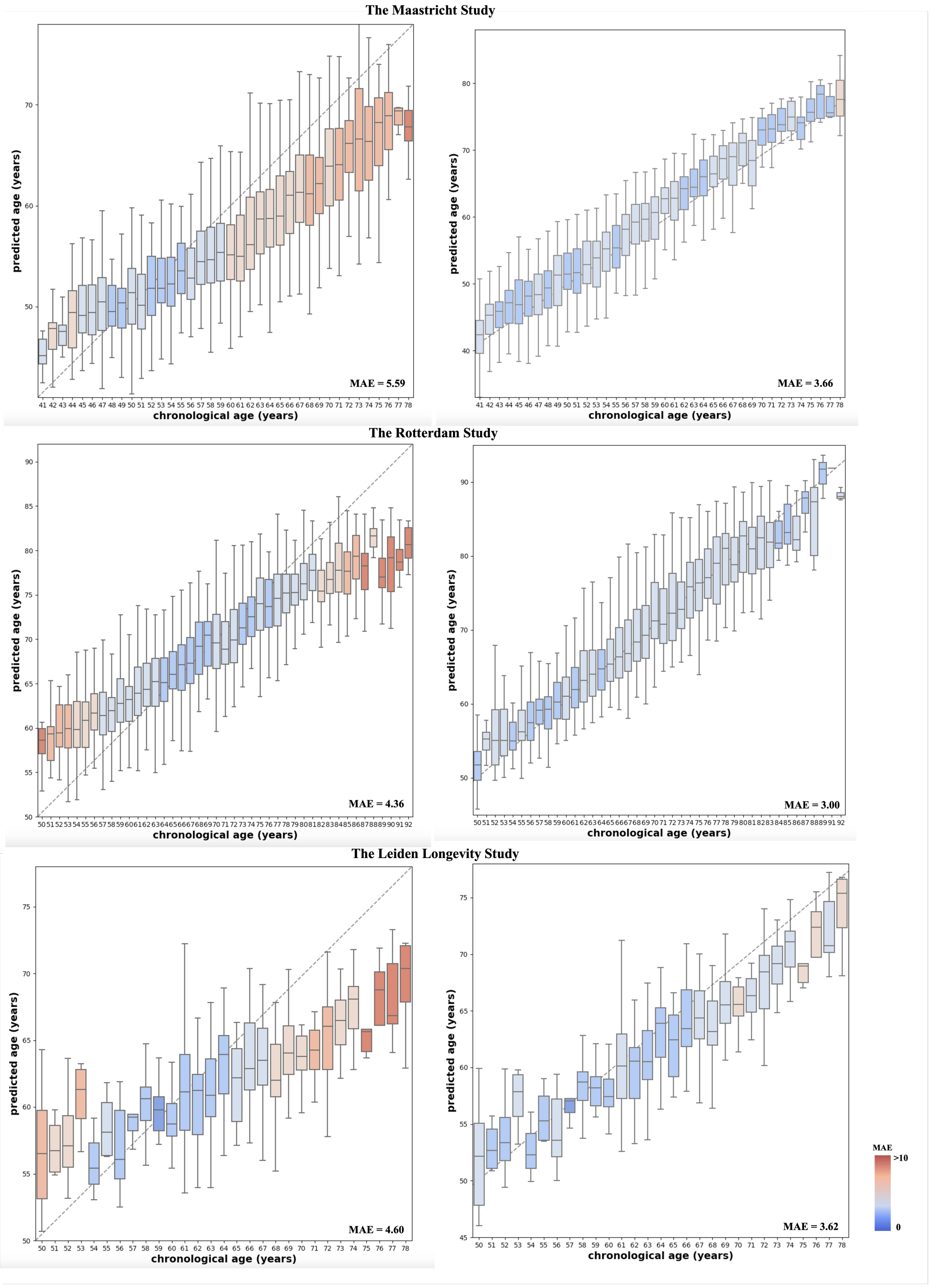

Fig. 2 Brain age prediction using the federated model (on the left) and applying the linear correction (on the right) for the test set of each cohort. Linear correction for TMS and RS was calculated in the respective training set, while LLS was estimated from both training cohorts (TMS and RS).

Table 4 3-fold cross-validation performance (MAE (range)) of BrainAge models trained locally and using the federated approach (test set).

	TMS	RS	Federated
TMS	4.67 (4.58, 4.73)	7.00 (6.22, 7.62)	6.34 (5.29, 7.00)
RS	7.88 (7.55, 8.46)	4.18 (4.16, 4.22)	4.68 (4.53, 4.95)
LLS	5.82 (5.10, 6.25)	5.23 (4.44, 6.32)	4.43 (4.00, 5.16)

Table 5 MAE of the federated BrainAge model trained with TMS and RS data when applying the age-bias correction models (test set). Three models were tested, one for each training set separately and one for the complete training set (TMS and RS). Values in brackets represent the 95% confidence interval.

	Without linear correction	With linear correction		
	-	TMS & RS	TMS	RS
TMS	5.59 [5.44, 5.76]	5.51 [5.37, 5.67]	3.66 [3.54, 3.77]	7.41 [7.25, 7.58]
RS	4.36 [4.21, 4.48]	3.33 [3.23, 3.44]	6.31 [6.17, 6.45]	3.00 [2.91, 3.08]
LLS	4.60 [4.25, 4.95]	3.62 [3.35, 3.88]	3.41 [3.13, 3.70]	4.73 [4.42, 5.03]

Table 6 MAE of the federated BrainAge model trained with TMS and RS data discretized by sex and diagnosis of diabetes. Values in brackets represent the 95% confidence interval.

	Sex		Diagnosis of diabetes	
	Male	Female	No	Yes
TMS	5.50 [5.29, 5.72]	5.68 [5.45, 5.94]	5.68 [5.48, 5.87]	5.19 [4.89, 5.50]
RS	4.43 [4.23, 4.63]	4.28 [4.11, 4.45]	4.31 [4.18, 4.45]	4.48 [4.09, 4.88]
LLS	4.64 [4.21, 5.12]	4.56 [4.07, 5.04]	4.67 [4.32, 5.07]	4.85 [3.17, 6.54]

3 The relation between BrainAge and MetaboAge

3.1 Full table of beta values including p-values and standard errors

Table 7 Beta values (beta), p-values (P) and standard errors (SE) for various levels of covariates for estimating MetaboAge (a) and MetaboHealth (b).

(a)

		M1	M2	M3	M4	M5	M6
	beta	0.16	-0.08	0.25	-0.01	0.22	-0.06
BrainAge	P	$4.3*10^{-32}$	$6.0*10^{-5}$	$2.2*10^{-66}$	$3.1*10^{-1}$	$2.3*10^{-49}$	$3.7*10^{-3}$
	SE	0.014	0.022	0.014	0.022	0.015	0.022
	beta		0.32		0.39		0.38
Age	P		$4.5 * 10^{-50}$		$1.8*10^{-65}$		$7.6*10^{-63}$
	SE		0.022		0.022		0.022
	beta			-0.16	-0.14	-0.27	-0.23
Sex	P			$1.2*10^{-17}$	$6.6*10^{-14}$	$1.5*10^{-27}$	$3.2*10^{-21}$
	SE			0.019	0.019	0.025	0.024
	beta			0.16	0.19	0.08	0.08
DM[1]	P			$3.25*10^{-6}$	$5.8*10^{-08}$	$2.1*10^{-2}$	$1.4*10^{-2}$
	SE			0.036	0.035	0.038	0.037
	beta			0.08	0.14	0.01	0.04
Lag Time	P			$2.5*10^{-9}$	$4.3*10^{-22}$	$2.1*10^{-1}$	$2.1 * 10^{-3}$
	SE			0.014	0.014	0.014	0.014
	beta					-0.03	-0.03
BMI	P					$1.5*10^{-2}$	$6.5*10^{-3}$
	SE					0.014	0.014
	beta					0.16	0.07
EC1[2]	P					$6.0*10^{-9}$	$4.4 * 10^{-3}$
	SE					0.028	0.028
	beta					0.11	0.11
EC3[2]	P					$1.6*10^{-5}$	$1.1*10^{-5}$
	SE					0.027	0.027
Error (MAE)		0.77	0.75	0.73	0.74	0.76	0.74

(b)

		M1	M2	M3	M4	M5	M6
	beta	0.13	0.11	0.13	0.10	0.10	0.09
BrainAge	P	$5.3*10^{-20}$	$1.7*10^{-6}$	$2.3*10^{-20}$	$2.1*10^{-6}$	$1.5*10^{-13}$	$1.4*10^{-5}$
	SE	0.014	0.022	0.014	0.022	0.014	0.022
	beta		0.03		0.02		0.06
Age	P		$9.3 * 10^{-2}$		$1.8*10^{-1}$		$2.1*10^{-3}$
	SE		0.022		0.022		0.022
	beta			-0.04	-0.04	-0.01	0.06
Sex	P			$8.6*10^{-3}$	$1.6*10^{-2}$	$2.9*10^{-1}$	$9.7*10^{-3}$
	SE			0.019	0.019	0.024	0.024
	beta			0.7	0.7	0.68	0.66
DM[1]	P			$9.07*10^{-86}$	$1.4*10^{-86}$	$1.9*10^{-73}$	$1.5*10^{-68}$
	SE			0.035	0.035	0.037	0.037
	beta			0.02	0.03	0.00	0.03
Lag Time	P			$6.6*10^{-2}$	$3.6*10^{-1}$	$2.1*10^{-1}$	$1.1 * 10^{-2}$
	SE			0.014	0.014	0.014	0.014
	beta					0.15	0.10
BMI	P					$1.2*10^{-26}$	$1.5*10^{-13}$
	SE					0.014	0.014
	beta					-0.07	-0.10
EC1[2]	P					$7.4*10^{-3}$	$2.4 * 10^{-4}$
	SE					0.027	0.028
	beta					-0.26	-0.28
EC3[2]	P					$9.1*10^{-23}$	$2.5*10^{-26}$
	SE					0.026	0.026
Error (MAE)		0.77	0.77	0.74	0.74	0.72	0.72

[1] DM = Diabetes Mellitus, i.e. diabetes (type 1 or 2) diagnosis.

[2] EC1-3 = Education Category, mapped to low/medium/high based on years of education. One-hot encoded relative to the medium level.

3.2 Comparison to meta-analytic framework

We compared the MataboAge regression model calculated through our iterative federated approach and those from a meta-analysis approach called HASE [32] (see Methods). The comparison between federated linear regression MAE's and HASE MAE's is in Table 8.

Table 8 Comparison between federated linear regression MAE's and HASE MAE's

Model	federated MAE	HASE MAE
M1	0.77	0.77
M2	0.75	0.75
M3	0.74	0.73
M4	0.74	0.73
M5	0.76	0.76
M6	0.76	0.76

Figure 3 presents a comparison between the beta values. Although most values are close, some outliers can be found in the categorical variables, being sex, diabetes diagnosis, and education category. However, when comparing mean absolute errors (Table 8), these differences seem to only make little impact.

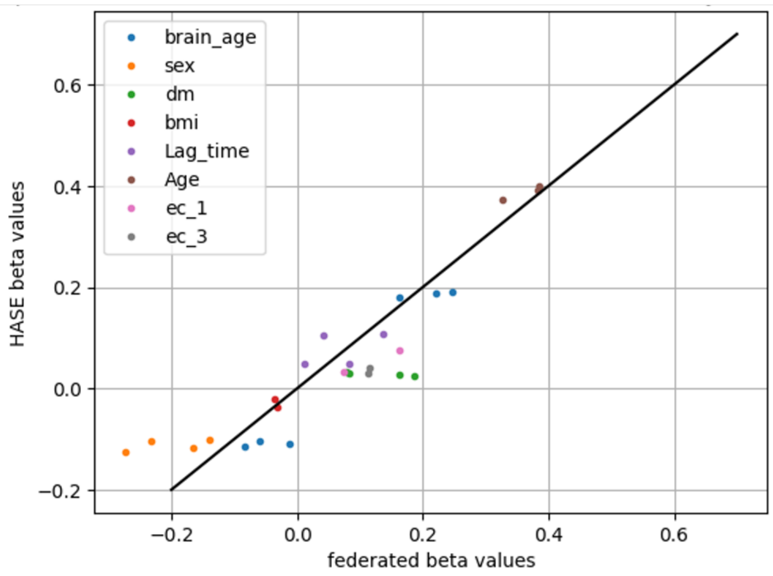

Fig. 3 Comparison of federated beta values with HASE beta values

4 Survival analysis

4.1 Survival analysis on dementia

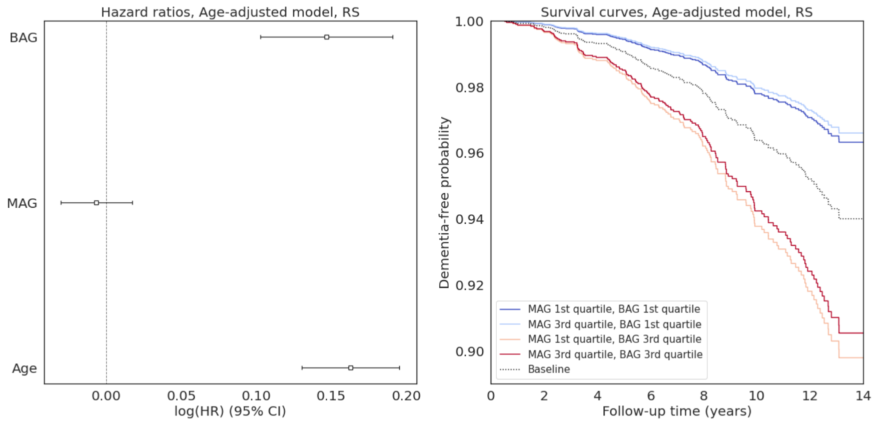

(a)

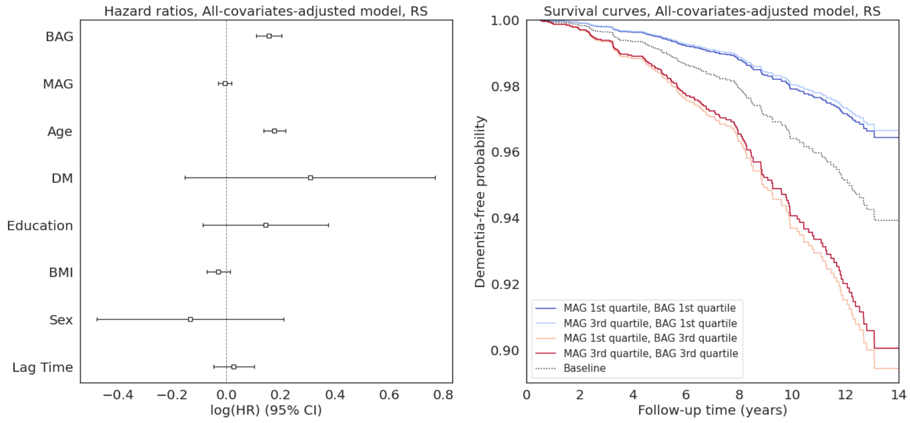

(b)

Fig. 4 Survival analysis for dementia prediction in RS using CPH models. On the left of (a) and (b) show the hazard ratios of the age-adjusted and all covariates-adjusted models, respectively. On the right show the survival curves of both models.

4.2 Survival analysis results using MetaboHealth

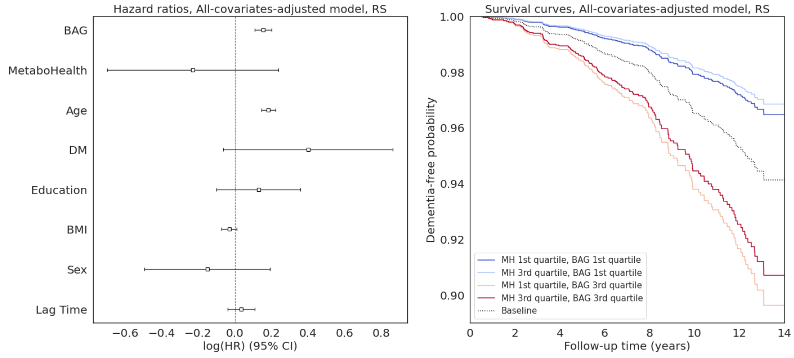

(a)

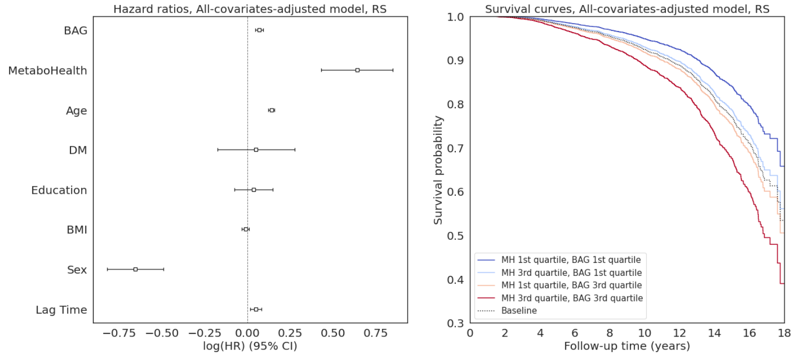

(b)

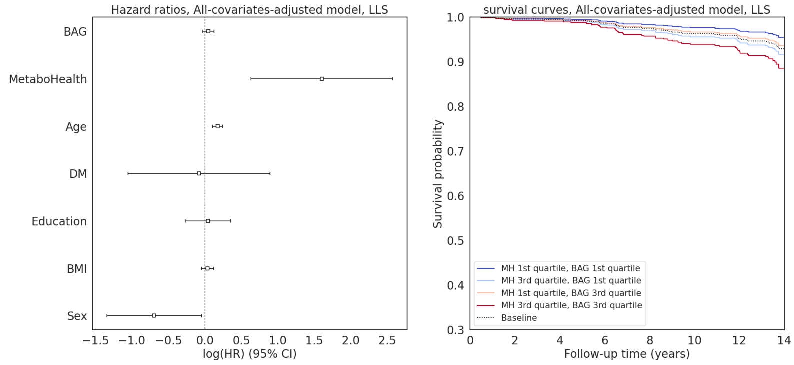

(c)

Fig. 5 Survival analysis results using MetaboHealth instead of MetaboAge, adjusting for age, diabetes mellitus (DM), education level, BMI and sex. (a) shows results for estimating dementia in RS, (b) and (c) show results for estimating mortality in RS and LLS, respectively.

4.3 Survival analysis results of separate and pairwise effects on BrainAge Gap, MetaboAge Gap, and MetaboHealth

Table 9 Survival analysis results of separate and pairwise effects on BrainAge Gap (BAG), MetaboAge Gap (MAG), and MetaboHealth in the Rotterdam Study (RS). Beta (log hazard ratios), p-values (P) and standard errors (SE) for covariates. (a) shows results for dementia prediction, and (b) for mortality prediction.

(a)

RS		M1	M2	M3	M4	M5	M6
	beta	0.15	-	-	0.16	0.16	0.16
BAG	P	$5.8*10^{-9}$	-	-	$4.7*10^{-11}$	$4.6*10^{-11}$	$3.9*10^{-11}$
	SE	0.03	-	-	0.02	0.02	0.02
	beta	-	0.01	-	-0.01	-	0.00
MAG	P	-	$5.1*10^{-1}$	-	$6.8*10^{-1}$	-	$9.1*10^{-1}$
	SE	-	0.01		0.01	-	0.01
	beta	-	-	-0.02	-	-0.23	-0.23
MetaboHealth	P	-	-	$9.4*10^{-1}$	-	$3.3*10^{-1}$	$3.6*10^{-1}$
	SE	-	-	0.23	-	0.24	0.26
	beta	0.20	0.08	0.07	0.18	0.18	0.18
Age	P	$8.0*10^{-31}$	$2.4*10^{-8}$	$4.2*10^{-11}$	$6.9*10^{-18}$	$5.3*10^{-21}$	$3.5*10^{-17}$
	SE	0.02	0.01	0.01	0.02	0.02	0.02
	beta	0.09	0.01	-0.01	-0.13	-0.15	-0.13
Sex	P	$6.4*10^{-1}$	$9.5*10^{-1}$	$9.6*10^{-1}$	$4.5*10^{-1}$	$3.9*10^{-1}$	$4.5*10^{-1}$
	SE	0.19	0.17	0.17	0.18	0.18	0.18
	beta	0.15	0.37	0.44	0.31	0.40	0.36
DM[1]	P	$6.4*10^{-1}$	$1.1*10^{-1}$	$5.8*10^{-2}$	$1.9*10^{-1}$	$9.1*10^{-2}$	$1.3*10^{-1}$
	SE	0.27	0.23	0.23	0.24	0.24	0.24
	beta	-	-	-	0.03	0.03	0.04
Lag Time	P	-	-	-	$4.8*10^{-1}$	$3.6*10^{-1}$	$3.5 * 10^{-1}$
	SE	-	-	-	0.04	0.04	0.04
	beta	-0.03	-0.02	-0.02	-0.03	-0.03	-0.03
BMI	P	$2.0*10^{-1}$	$3.5*10^{-1}$	$3.1*10^{-1}$	$1.7*10^{-1}$	$1.5*10^{-1}$	$2.0*10^{-1}$
	SE	0.02	0.02	0.02	0.02	0.02	0.02
	beta	0.28	0.09	0.08	0.14	0.13	0.14
Education	P	$2.4*10^{-2}$	$4.1*10^{-1}$	$3.9*10^{-1}$	$4.7*10^{-1}$	$2.7*10^{-1}$	$2.2 * 10^{-1}$
	SE	0.12	0.12	0.12	0.12	0.12	0.12

[1] DM = Diabetes Mellitus, i.e. diabetes (type 1 or 2) diagnosis.

(b)

RS		M1	M2	M3	M4	M5	M6
	beta	0.08	-	-	0.07	0.07	0.07
BAG	P	$5.6*10^{-12}$	-	-	$6.2*10^{-10}$	$1.7*10^{-9}$	$1.7*10^{-9}$
	SE	0.01	-	-	0.01	0.01	0.01
	beta	-	0.02	-	0.01	-	0.01
MAG	P	-	$2.2*10^{-4}$	-	$1.9*10^{-2}$	-	$4.1*10^{-1}$
	SE	-	0.01	-	0.01	-	0.01
	beta	-	-	0.77	-	0.64	0.65
MetaboHealth	P	-	-	$1.4*10^{-13}$	-	$2.2*10^{-9}$	$1.7*10^{-8}$
	SE	-	-	0.10	-	0.11	0.11
	beta	0.16	0.09	0.06	0.16	0.14	0.14
Age	P	$1.4*10^{-111}$	$3.4*10^{-32}$	$4.5*10^{-33}$	$7.9*10^{-57}$	$3.4*10^{-53}$	$2.1*10^{-45}$
	SE	0.01	0.01	0.01	0.01	0.01	0.01
	beta	-0.63	-0.60	-0.59	-0.66	-0.66	-0.68
Sex	P	$2.6*10^{-14}$	$3.4*10^{-13}$	$1.1*10^{-12}$	$2.8*10^{-15}$	$3.8*10^{-15}$	$7.1*10^{-16}$
	SE	0.08	0.08	0.08	0.08	0.08	0.08
	beta	0.21	0.19	0.05	0.05	0.18	0.03
DM[1]	P	$5.8*10^{-2}$	$8.9*10^{-2}$	$6.6*10^{-1}$	$1.0*10^{-1}$	$6.7*10^{-1}$	$7.8*10^{-1}$
	SE	0.11	0.11	0.11	0.11	0.11	0.12
	beta	-	-	-	0.08	0.05	0.05
Lag Time	P	-	-	-	$8.0*10^{-6}$	$4.3*10^{-3}$	$3.0 * 10^{-3}$
	SE	-	-	-	0.02	0.02	0.02
	beta	-0.01	0.00	-0.01	0.00	-0.01	-0.01
BMI	P	$5.6*10^{-1}$	$1.0*10^{0}$	$4.0*10^{-1}$	$7.3*10^{-1}$	$2.5*10^{-1}$	$3.3*10^{-1}$
	SE	0.01	0.01	0.01	0.01	0.01	0.01
	beta	0.03	0.01	0.02	0.03	0.04	0.04
Education	P	$5.9*10^{-1}$	$9.1*10^{-1}$	$7.8*10^{-1}$	$5.9*10^{-1}$	$5.3*10^{-1}$	$4.4*10^{-1}$
	SE	0.06	0.06	0.06	0.06	0.06	0.06

Table 10 Survival analysis results of separate and pairwise effects on BrainAge Gap (BAG),MetaboAge Gap (MAG), and MetaboHealth on mortality prediction for the Leiden Longevity Study (LLS).

LLS		M1	M2	M3	M4	M5	M6
BAG	beta	0.07	-	-	0.07	0.05	0.05
	P	$7.9 * 10^{-2}$	-	-	$7.9 * 10^{-2}$	$2.5 * 10^{-1}$	$2.4 * 10^{-1}$
	SE	0.04	-	-	0.04	0.04	0.04
MAG	beta	-	0.03	-	0.03	-	0.02
	P	-	$2.1 * 10^{-1}$	-	$2.1 * 10^{-1}$	-	$3.5 * 10^{-1}$
	SE	-	0.02	-	0.02	-	0.02
MetaboHealth	beta	-	-	1.7	-	1.6	1.6
	P	-	-	$4.1 * 10^{-4}$	-	$1.2 * 10^{-3}$	$1.8 * 10^{-3}$
	SE	-	-	0.49	-	0.50	0.50
Age	beta	0.19	0.18	0.15	0.22	0.17	0.19
	P	$5.1 * 10^{-8}$	$3.2 * 10^{-7}$	$3.5 * 10^{-7}$	$6.0 * 10^{-8}$	$9.4 * 10^{-7}$	$1.0 * 10^{-6}$
	SE	0.04	0.04	0.03	0.04	0.04	0.04
Sex	beta	-0.73	-0.75	-0.74	-0.69	-0.69	-0.67
	P	$2.5 * 10^{-2}$	$2.2 * 10^{-2}$	$2.4 * 10^{-2}$	$3.5 * 10^{-2}$	$3.5 * 10^{-2}$	$4.3 * 10^{-2}$
	SE	0.32	0.33	0.33	0.33	0.33	0.33
DM[1]	beta	0.39	0.40	-0.12	0.49	-0.08	0.05
	P	$4.1 * 10^{-1}$	$4.1 * 10^{-1}$	$8.0 * 10^{-1}$	$3.2 * 10^{-1}$	$8.7 * 10^{-1}$	$9.3 * 10^{-1}$
	SE	0.48	0.48	0.49	0.49	0.50	0.51
Lag Time	beta	-	-	-	-	-	-
	P	-	-	-	-	-	-
	SE	-	-	-	-	-	-
BMI	beta	0.05	0.07	0.05	0.05	0.04	0.04
	P	$2.3 * 10^{-1}$	$8.9 * 10^{-2}$	$2.5 * 10^{-1}$	$2.3 * 10^{-1}$	$3.9 * 10^{-1}$	$3.7 * 10^{-1}$
	SE	0.04	0.04	0.04	0.04	0.04	0.04
Education	beta	0.02	0.02	0.03	0.04	0.04	0.06
	P	$9.0 * 10^{-1}$	$9.2 * 10^{-1}$	$8.4 * 10^{-1}$	$7.8 * 10^{-1}$	$7.9 * 10^{-1}$	$7.0 * 10^{-1}$
	SE	0.16	0.16	0.16	0.16	0.16	0.16

References

[1] P. Mateus, J. Moonen, M. Beran, *et al.*, "Data harmonization and federated learning for multi-cohort dementia research using the omop common data model: A netherlands consortium of dementia cohorts case study," *Journal of Biomedical Informatics*, p. 104 661, 2024, ISSN: 1532-0464. DOI: https://doi.org/10.1016/j.jbi.2024.104661.

[2] D. S. Marcus, T. R. Olsen, M. Ramaratnam, and R. L. Buckner, "The extensible neuroimaging archive toolkit: An informatics platform for managing, exploring, and sharing neuroimaging data," *Neuroinformatics*, vol. 5, pp. 11–33, 2007.

[3] R. De Boer, H. A. Vrooman, F. Van Der Lijn, *et al.*, "White matter lesion extension to automatic brain tissue segmentation on mri," *Neuroimage*, vol. 45, no. 4, pp. 1151–1161, 2009.

[4] J. Monereo-Sánchez, J. J. de Jong, G. S. Drenthen, *et al.*, "Quality control strategies for brain mri segmentation and parcellation: Practical approaches and recommendations-insights from the maastricht study," *Neuroimage*, vol. 237, p. 118 174, 2021.
